# A New Method of Intestinal Anastomosis

**Published:** 1898-10

**Authors:** 


### Abstracts and Selections.

### A New Method of Intestinal Anastomosis.

The possibilities in surgery, and especially in abdominal surgery, appear to be practically illimitable. But a few years ago, and the prediction that the operation for removing the entire stomach could be performed with any degree of success would have been scouted as the vaporings of a dreamer. Now that this apparently impossible feat has become an accomplished fact, it would be rash to assert that any operation, however radical, is too difficult for the surgeon to attempt with the hope of good results. This advance in practical surgery, in which our country has played a satisfactory part, is undoubtedly one of the most striking features of recent years. Almost every week some novel mode of performing an operation is suggested or tried. This is as it should be, for it is in experiments that the path which leads to final success lies. Dr. J. Shelton Horsley, in the *New York Polyclinic* for December, describes a new method of intestinal anastomosis that has occurred to him, and which has been tested on animals and the cadaver. From a perusal of this article, it would appear that the means proposed by Dr. Horsley are both feasible and simple. These are as follows: "After the incision in the abdominal wall, the diseased intestine is drawn well out of the abdominal cavity, and sterilized tapes are placed through the mesentery several inches from the point to be resected, and tied round the intestine just tightly enough to check the fæcal flow. Fæcal matter is stripped from between the tapes before they are tied. The intestine is resected, bleeding po nts in the mesentery are secured with artery forceps and ligated with silk, and the ends mopped out with moist bichloride sponges. The mesentery is incised for several inches at right angles to the intestine, or a V-shaped section removed to facilitate the subsequent manipulation. The ends of the bowel are then placed side by side, opening in the same direction and being in contact along their free surfaces opposite the attached mesentery. A pair of artery forceps inserted in the ends and clamped holds them in this position. A finger of the left hand is inserted into one end of the intestine and the thumb into the other, and over them as a bobbin a Cushing suture of fine silk in an ordinary cambric needle is commenced. The first stitch approximates the portion of the two limbs of the intestine near the mesenteric attachment. The suture is then carried obliquely for about two inches when dealing with the small intestine, to the border opposite the mesenteric attachment, and continued over the other side, where it stops at a place corresponding to its point of commencement. Here the needle is left on the thread, and an artery forceps, padded with sterilized gauze to prevent injury to the thread, seizes it where it emerges at the last stitch. This keeps the sutures tight. The bowel is now partly everted, exposing a U-shaped septum, grasped by the artery forceps first applied. This septum is cut away with curved scissors, leaving a margin of one-third of an inch. An overhand suture of silk in a curved needle is then commenced at one edge of the 'shelf' left by cutting away the septum, and is carried through all the intestinal coats. When the suture reaches the end of this shelf, it is continued by slightly invaginating the rest of the resected ends, which consists of about one-fourth of the entire circumference. It terminates at its point of commencement. The first line of sutures is now finished by continuing it about one-fourth of an inch from the overhand suture. The incision in the mesentery is closed, the intestine lightly sponged with gauze wrung out of hot sterilized salt solution, and dropped back into the abdominal cavity."

It is claimed as a special feature of this manner of operating that all fear of stricture is removed. Experiments made on three dogs by Dr. Horsley resulted as he had anticipated.—*New York Medical Record*, February 12, 1898.

